# Modalities of group A streptococcal prevention and treatment and their economic justification

**DOI:** 10.1038/s41541-023-00649-3

**Published:** 2023-04-22

**Authors:** Jeffrey W. Cannon, Rosemary Wyber

**Affiliations:** 1 Wesfarmers Centre of Vaccines and Infectious Diseases, Telethon Kids Institute, Nedlands, WA Australia; 2 Department of Global Health and Population, Harvard T.H. Chan School of Public Health, Boston, MA USA; 3 National Centre for Aboriginal and Torres Strait Islander Wellbeing Research, National Centre for Epidemiology and Population Health, ANU College of Health & Medicine, The Australian National University, Canberra, Australia; 4 Adjunct Senior Research Fellow, University of Western Australia, Nedlands, WA Australia

**Keywords:** Bacterial infection, Public health

## Abstract

Infection by group A *Streptococcus* (Strep A) results in a diverse range of clinical conditions, including pharyngitis, impetigo, cellulitis, necrotising fasciitis, and rheumatic heart disease. In this article, we outline the recommended strategies for Strep A treatment and prevention and review the literature for economic evaluations of competing treatment and prevention strategies. We find that most economic evaluations focus on reducing the duration of illness or risk of rheumatic fever among people presenting with sore throat through diagnostic and/or treatment strategies. Few studies have evaluated strategies to reduce the burden of Strep A infection among the general population, nor have they considered the local capacity to finance and implement strategies. Evaluation of validated costs and consequences for a more diverse range of Strep A interventions are needed to ensure policies maximise patient outcomes under budget constraints. This should include attention to basic public health strategies and emerging strategies such as vaccination.

## Introduction

Infection by *Streptococcus pyogenes* (group A *Streptococcus*, or ‘Strep A’) can cause a wide spectrum of clinical manifestations. Superficial infections of the upper respiratory tract and skin can lead to pharyngitis and impetigo, respectively, as well as blood stream and other sterile site infections, manifesting as necrotising fasciitis, toxic shock syndrome, sepsis, maternal sepsis, osteomyelitis, and meningitis. Strep A infection can also cause the immune-mediated sequelae of acute rheumatic fever (ARF) and acute post-streptococcal glomerulonephritis (APSGN), which in turn can lead to chronic rheumatic heart disease (RHD) and chronic kidney disease (CKD)^1,2^ respectively.

Collectively, Strep A infections and complications cause over half a million deaths globally each year, making it—before the emergence of SarS-COVID-2—the fifth most lethal pathogen on the planet behind *HIV, Mycobacterium, tuberculosis, Plasmodium falciparum*, and *Streptococcus pneumoniae*^3^. The morbidity and mortality from Strep A infection is inequitably concentrated in resource-limited settings, though the burden of sepsis and superficial infections is also significant in high-resource settings. For example, sore throat is the third most common diagnosis with an antibiotic prescription in the U.S. and the most common reason for antibiotic self-medication in Europe^4,5^.

The protracted etiological pathway from superficial Strep A infection to chronic sequelae or death present a wide range of potential opportunities for therapeutic intervention. For the prevention of ARF and RHD, interventions are generally codified into four broad categories: primordial, primary, secondary, and tertiary prevention. Primordial prevention traditionally focuses on preventing infection, and therefore ARF and RHD, by reducing Strep A exposure and transmission through hygiene and social distancing^6^. Primary prevention is treatment of superficial infection and is intended to minimize the risk of ARF. Secondary prevention is antibiotic prophylaxis intended to prevent infection and recurrent ARF in people with a history of ARF or RHD^7^. Tertiary prevention is intended to improve duration or quality of life through medical or surgical management, such a heart valve surgery for people with severe RHD.

The technology for almost all these Strep A mitigation strategies has existed for decades. However, there is little consensus about how clinicians or policy-makers in different settings should choose between different strategies. Nor is there guidance about how to consider potentially transformative technology, such as development of a Strep A vaccine for primordial prevention. Economic modelling can support decision making on these issues by outlining the costs and benefits of different Strep A control strategies. Economic models are most useful to decision makers when they are clear about their scope, data inputs, assumptions, uncertainties, and limitations. However, clarity on these issues is more achievable for near-term clinical therapies than for long-term or large-scale public health strategies. This may lead to systematic bias if strategies that are readily evaluated are published more than strategies that are uncertain or complex. Selection and publication bias is particularly significant for Strep A because of the diversity of clinical manifestations and their variable latency (spanning weeks, months, or years), as well as the breadth of potential interventions. These factors also amplify the interactions and externalities of Strep A control strategies; for example, primary prevention may have a measurable near-term impact on morbidity but a less-certain, long-term impact on incident chronic kidney disease. Therefore, policy makers addressing Strep A need access to both economic evaluation of individual strategies and to contextual analysis of how each approach fits into broader disease control goals and opportunities.

This review compiles evaluations of Strep A treatment or prevention strategies that included an economic analysis. Categorising these evaluations according to an agreed framework makes it possible to identify which Strep A control strategies have the most robust economic evaluations and which strategies have been under-explored. Identifying evaluation gaps provides important context for policy makers and should inform a research agenda to better support decision-makers in the future. These goals are particularly important in mitigating the significant global burden of Strep A as policy makers consider new strategies—such as Strep A vaccination—relative to existing technologies.

## Results

**Fig. 1:**
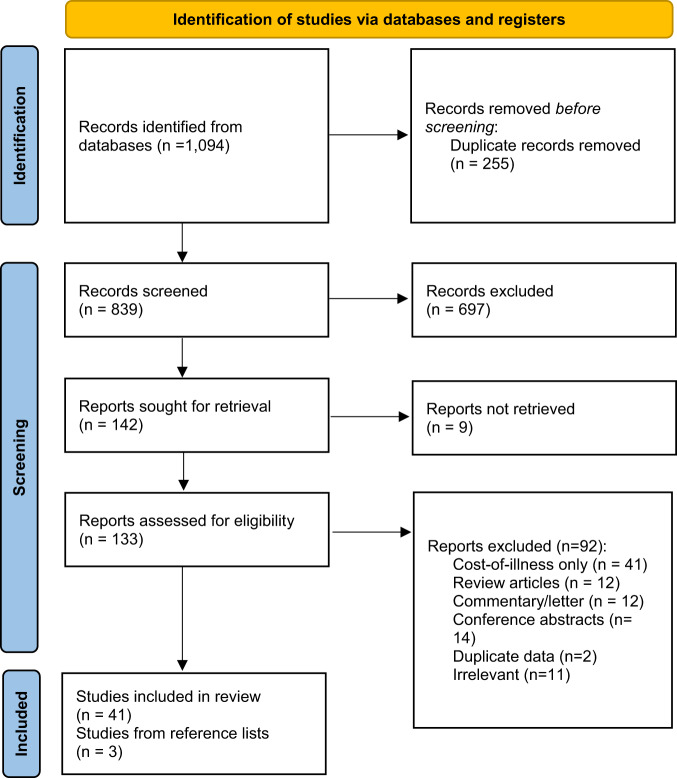
PRISMA 2020 flow diagram for new systematic reviews which included searches of databases and registers only. Source: Page et al.^72^.

We identified 839 studies after removing 255 duplicate studies (Fig. 1). Of those, 142 studies were considered for full-text review, but we were unable to locate the full-text for nine studies^8–16^. After full-text review of 133 studies, 92 studies were excluded and 41 studies were included in our review. In addition, we included another three studies that were found from reviewing the reference lists of the 41 eligible studies. Thus, 44 studies were included for analysis, each of which conducted an economic evaluation of Strep A treatment or prevention strategies.

Studies included for analysis are summarised by prevention category in Supplementary Table 1. Of the 44 studies, primordial prevention was evaluated in one study, primary prevention in 34 studies, secondary prevention in eight studies, and tertiary prevention in one study. Six of the 44 studies compared strategies across multiple categories.

### Primordial prevention

One study, from Australia, evaluated the potential value of vaccines against Strep A infection from a health sector perspective (Supplementary Table 2)^17^. Based on assumed levels of vaccine efficacy, coverage, and durability, the study estimated that annual vaccination campaigns costing less than AU$260–289 per non-Indigenous and AU$897–920 per Indigenous child would be considered to have an acceptable incremental cost-effectiveness ratio compared to no vaccination. At a population level, reductions in the incidence of superficial throat and skin diseases and cellulitis added considerable value to vaccines.

### Primary prevention: reduced duration of illness without evaluating prevention of sequelae

There were 16 studies that included an economic evaluation of strategies to manage throat, skin, or soft tissue infection but that did not include the benefits of preventing down-stream complications (e.g., quinsy, ARF, APSGN; Supplementary Table 3). Of the 16 studies, six were related to pharyngitis, two to impetigo, seven to cellulitis, and one to general skin and soft tissue infections.

All the studies on managing pharyngitis were conducted in upper-middle or high-income countries. One study evaluated treatment choice^18^ and five evaluated diagnostic strategy^19–23^. Four of the five studies that evaluated diagnostic strategies concluded that routine use of rapid antigen detection tests (RADTs) resulted in the lowest cost per case of accurate diagnosis and antibiotic treatment^19–22^. The other study evaluated backup testing for negative RADTs, concluding that it was of limited value^23^.

Half of studies on primary prevention for skin infections evaluated antibiotic, analgesic or adjunct treatment choices. This included both studies for impetigo^24,25^, two of the seven studies for cellulitis^26,27^, and the two studies on general skin and soft tissue infections (SSTIs)^28,29^. The remaining five studies for cellulitis evaluated management options or treatment setting^30–34^.

### Primary prevention: treat acute disease to reduce duration of illness and prevent ARF or other complications of infection

Six studies evaluated diagnostic strategies for patients presenting for treatment of sore throat. All included the benefits of reducing duration of acute illness and preventing ARF and/or quinsy (Supplementary Table 4). In addition, some studies included further down-stream benefits, namely preventing RHD due to preventing ARF.

The earliest of those studies, a study by Neuner et al. published in 2003^35^, was a model-based, cost-utility analysis for pharyngitis treatment among adult patients from a societal perspective. The authors estimated the costs and quality-adjusted life-days (QLADs) lost due to pharyngitis and related complications for five diagnosis and treatment strategies: observation without testing or treatment, empirical treatment with penicillin, throat culture all patients and treat Strep A positive cases, RADT for all patients followed by culture to confirm negative test, and RADT without follow-up culture for all patients. They concluded that performing a throat culture on all patients and treating Strep A positive cases was the least costly and the most effective (least QALDs lost to pharyngitis and complications) strategy. From sensitivity analyses, the authors conclude that observation without testing or treatment would be more effective if the probability of ARF was lower (baseline was 5 in 10,000 adults with Strep A pharyngitis), the probability of anaphylaxis was higher, or the quality of life with pharyngitis was better compared to baseline values. These results are important as there was uncertainty in all parameter estimates, which were derived from few and aging studies from the literature or from unsubstantiated assumptions.

Subsequently, Van Howe et al.^36^ performed a similar analysis to Neuner et al. using many of the same parameter values, but their evaluation was among children rather than adult patients. Three variations to the parameter values used by Neuner et al. were: a considerably lower risk of ARF (7 in 1,000,000 children with Strep A pharyngitis); a higher prevalence of Strep A pharyngitis; and analysis of different costs for throat culture and RADT, which depended on payer perspective (private vs. Medicaid prices). They conclude that when the cost of a throat culture is similar to the cost of a RADT (i.e., US Medicaid perspective), then performing a culture on all patients is cost-effective. When the cost of culture increases relative to RADT (i.e., private perspective), then RADT is the cost-effective strategy. Sensitivity analysis indicated that the risks of ARF and quinsy had the most impact on results. The sensitivity analyses findings are important because the study authors estimated a lower risk of ARF in children with Strep A pharyngitis compared to the risk that was used among adult patients in the study by Neuner et al.^35^, yet the incidence rates of Strep A pharyngitis and ARF are higher in children.

Klepser et al.^37^ preformed a similar study to Neuner et al. but added a strategy of diagnosis and treatment at a pharmacy, which they found to be cost-effective. Behnamfar et al.^38^ conclude that RADT is cost effective compared to other diagnostic strategies, but their level and source of effectiveness was not described.

Little et al.^39^, in 2014, present the only trial-based evaluation of diagnostic strategies and collect and report primary data on quality-adjusted life-years (QALYs), which were calculated from EuroQol EQ-5D scores^40^ measured during the trial. The aggregate QALYs reflected morbidity due to sore throat and a low risk of quinsy, but it did not include morbidity due to ARF as there were no cases observed during the trial. Fraser et al.^41^ extended, through a model-based study, the evaluation of Little et al. to include the costs and benefits of preventing ARF by incorporating relevant data from Neuner et al.^35^ and to include the test characteristics of all commercially available RADTs through sensitivity analyses. From each study, Little et al. recommend diagnosis by clinical decision rule (CDR; in line with Neuner’s sensitivity analysis of low ARF risk)^39^, and Fraser et al. did not find RADT testing, compared to no testing, among patients with a high CDR score to be cost-effective^41^.

### Primary prevention: primary prevention without reducing duration of infection

Five studies evaluated primary prevention strategies based on preventing ARF and/or quinsy but not the benefit of reduced duration of acute illness (Supplementary Table 5). Of those, three were cost-effectiveness analyses (CEAs), which used a cost per averted compilation as the analysis outcome, and two were cost-utility analyses (CUAs). Of the two CUAs, one evaluated genetic testing for ARF susceptibility at birth^42^, and the evaluated methods to diagnose Strep A infection, not including RADT, among pharyngitis patients^43^. All three CEAs, which were conducted among HICs, found that performing an RADT on all patients or performing an RADT on patients with a high Clinical Decision Rule (CDR) score were cost-effective strategies for preventing complication of Strep A pharyngitis^44–46^; the study that found RADT on patients with a high CDR also included complications of antibiotic therapy in its measure of effectiveness^45^.

### Primary prevention: primary and other prevention categories

Six studies evaluated down-stream strategies in addition to primary prevention, two of which combined the down-stream strategies in an overall program and four evaluated them as independent strategies (Supplementary Table 6). Both of the two studies evaluating a combined program of interventions, which were public health interventions rolled out in Cuba between 1986 and 2002, concluded that they were economically efficient compared to the previous healthcare measures^47,48^.

Among the four studies evaluating strategies independently, three were set in African countries and the other was in India. Of the evaluations focused in Africa, one found that primary prevention was most cost-effective and two found that secondary and tertiary prevention was cost-effective. The study that found primary prevention to be cost-effective included improving health-seeking behaviours for pharyngitis and was based on preventing only new cases of ARF/RHD^49^. However, that study aimed to demonstrate the application of a model as a tool to evaluate ARF/RHD prevention strategies rather than exhaustively evaluate specific strategies. One of the two studies that found secondary prevention to be cost-effective did not include improving rates of health seeking behaviour for pharyngitis treatment^50^. The study found that providing secondary prophylaxis in children with echocardiographic-detected RHD was cost-effective compared to doing nothing, while providing long-term antibiotic prophylaxis to all children regardless of clinical history or providing treatment for presenting pharyngitis patients with a culture positive test was not cost-effective compared to the echo screening strategy. The other study found secondary and tertiary prevention was a net cost-benefit, but unlike the previous two studies, it included prevention of adverse outcomes in both incident and prevalent cases^51^. The study from India, which was also based on preventing the burden of disease among only incidence cases, found that both primary and secondary prevention had net benefits that outweighed costs, but the benefit was higher for primary prevention^52^.

### Secondary and tertiary prevention

Five studies evaluated secondary or tertiary prevention strategies among people with a history of ARF or RHD (Supplementary Table 7). One study focused solely on improving patients’ adherence to monthly penicillin infection^53^. The authors reported a cost per extra BPG injection, with ‘cost-effectiveness’ to be decided by the policy maker. It did not quantify the benefits of the intervention beyond improving compliance.

Three studies, all conducted during the advance of portable echo screening devices, concluded that echo screening was cost-effective compared to existing care^54–56^. While there was a major limitation in evidence of effectiveness (i.e., no evidence as no clinical trials or evaluations had been conducted to evaluate the outcomes of patients detected by routine screening compared to those diagnosed through usual care), sensitivity analyses demonstrated conditions (e.g., incidence rates, screening test characteristics, costs) under which screening would not be considered cost-effective, which may be useful for future studies aiming to collect key data.

One study set in India evaluated surgical interventions for rheumatic mitral valve disease patients aged 20 and over^57^. Repair for all patients was cost-effective compared to usual care (mixture of procedures) or replacement for all patients.

In addition, four studies evaluated the prevention of recurrent disease other than ARF (Supplementary Table 8). Two of those studies evaluated tonsillectomy as a prevention strategy for frequently recurring pharyngitis^58,59^, and they both found that tonsillectomy was cost-effective compared to usual care. One study found that homeopathic remedy (SilAtro-5–90) as an adjuvant to normal treatment was cost-effective in preventing recurrent pharyngitis^60^. Lastly, a study evaluating strategies to prevent recurrent cellulitis found that antibiotic prophylaxis was not associated with a significant increase in costs but reduced the risk of recurrence by nearly a third^61^.

### Evaluation quality

An assessment of each economic evaluation against Drummond et al.’s checklist is presented in Supplementary Table 9. Over half of the studies (27 out of 44 studies) scored eight or more out of 10 and a score of six or more was achieved in 35 studies. The most common items missing from the evaluations were a stated perspective of the evaluation (18 studies) and, largely as a consequence of an unclear perspective, the identification of all important and relevant costs and consequences (18 studies). Other common items absent were an incremental analysis (the difference in costs compared to the difference in benefits between strategies; 15 studies), a discussion of implementation issues or of similar evaluations (14 studies), and the establishment of intervention effectiveness (12 studies).

## Discussion

This review synthesises two decades of studies incorporating economic evaluation of Strep A prevention and treatment modalities. The cohort of these studies are notable for their scope. Though, the vast majority evaluated clinical diagnostic or therapeutic strategies to manage acute disease and focused on near-term outcomes. For example, the largest number of included publications addressed primary prevention (*n* = 19), particularly diagnostic strategy for acute pharyngitis (*n* = 5). These studies largely found that performing RADTs in all patients presenting with pharyngitis was cost-effective in reducing antibiotic consumption compared to other diagnostic and management strategies. Use of RADT was also a favourable strategy for diagnosing and managing pharyngitis when considering the reduction in risk of ARF and other sequelae in studies that did, and did not, include reductions in duration of infection as a benefit. However, few, if any, studies considered the implications of diagnostic tests detecting upper respiratory tract carriage of Strep A among cases of viral infection, which might lead to unnecessary antibiotic treatment.

The number of studies focusing on diagnostic strategies may suggest to decision-makers that this is the key clinical or public health question in addressing Strep A burden; i.e., the area with the most evaluation is the impost important or impactful. However, studies which identify cost-effective strategies for pharyngitis management do not necessarily translate into studies for cost-effective strategies to reduce the population-level burden of post-infection sequalae, including ARF and RHD. Only seven studies evaluated strategies to reduce the incidence of ARF and/or RHD among the general population^17,47–52^, and four studies evaluated strategies to prevent worsening of established disease^54–57^. While these studies indicate that improving the coverage of existing or introducing new prevention strategies are economically justifiable, the body of existing literature does not clearly meet the needs of decision makers targeting the population-wide reduction in post-infection sequelae.

Supporting decision makers with clear, economic justification for strategies to prevent the burden of Strap A infection is hindered by a range of issues. First, almost half of the economic evaluations did not state the perspective of the analysis. The analysis perspective defines the key decision maker, or payer, that the evaluation is aimed at, the related costs and consequences included in the analysis, and other factors^62^. Analyses from a healthcare sector perspective would include all costs and consequences relevant to,for example, a health minister when allocating public funding to health technologies and services, while a societal perspective would include a broader range of costs and consequences relevant to, for example, a finance ministerwho considers broader health, economic, and social outcomes. Because of ill-defined perspective, and possibly differences in treatment guidelines between regions, the resources required to implement interventions and manage disease varied across studies. Second, the impact of post infectious sequala on quality-of-life within a health economic framework (e.g., QALY weights) have never been measured or validated. This is particularly important for ARF or RHD as diagnosis of these condition implies up to a decade of prophylaxis, which is recognised to be painful and have significant administrative burdens. Third, evaluations focused on primary prevention of ARF alone did not include the benefit of reducing duration of acute illness through appropriate antibiotic treatment. Given the frequency of sore throat compared to ARF, reducing duration of illness may be an important factor in the economic case for primary prevention. Forth, many evaluations considered population-level interventions and, therefore, missed the differential impacts on groups with variable risks within those populations. Indigenous and other minority groups that are marginalised in several high-income countries have high rates of severe invasive infections and ARF and RHD^63^. These groups can have diagnostic and treatment strategies that differ to the rest of the population, and economic evaluations should be conducted separately for these risk-based strategies.

Decision making may also be hindered by epidemiological uncertainties and inconsistencies between Strep A disease models and observational studies, as well as lacking evidence on the effectiveness of several evaluated strategies. The majority of evaluations were model-based evaluations, which used arguably outdated data or assumptions for parameter estimates. This included the risk of ARF after an untreated Strep A infection, which ranged from 0.00007 cases of ARF per 100^36^ untreated Strep A infections to approximately 2 cases of ARF^52^ or 2 cases of RHD^50^ per 100 untreated Strep A infections across evaluations of pharyngitis management and ARF prevention strategies. Further, strategies for the primary prevention of ARF and RHD did not include the treatment or prevention of impetigo as a risk factor, yet an increasing amount of studies during the period of this review have indicated that impetigo is likely to be implicated in ARF pathogenesis^64,65^. Several studies made assumptions around the effectiveness of the prevention strategy, primarily strategies that are hindered, in part, by the expense of evaluating them by clinical trials. While this is not an exhaustive list, there is a clear need for new primary research to improve model certainty in modelled outcomes and utility for people relying on them for decision making.

The absence of studies addressing key issues in Strep A control was revealing. For example, there were no economic evaluations for primordial prevention of infection by addressing environmental or social factors or hygiene infrastructure or behaviours. Nor were there evaluations of tertiary prevention of invasive infection (i.e., adjuvant therapy). No evaluations addressed all strategies along the aetiological pathway between infection and severe disease as independent or combined strategies. One cost-of-illness study estimated reductions in direct healthcare expenditure due to primordial prevention^6^; estimates for these costs were presented but not formally contrasted against the costs of the interventions. On inspection, the intervention costs dwarf the cost savings related to ARF/RHD prevention. However, interventions which address social determinants of health are likely to result in health and economic benefits for other infectious disease, as well as a broader range of economic benefits, such as improved educations and productivity. Of note however, most studies used a human capital approach to estimating productivity gains, whereas a frictional cost approach, particularly in the context of strategy affordability, may be more suited among settings where disease prevention does not necessarily translate into an increased and fully employed labour force. This illustrates an important unmet need to comprehensively evaluate a wider range of health, economic, and social benefits associated with public health strategies, rather than attempting to quantify the potential benefits for single disease endpoints^66^.

Few studies outlined or analysed the capacity of the health system to implement recommended strategies in regard to their affordability or specialist workforce requirements, which is critical information for decision makers, particularly in resource-constrained settings. For example, short-term financial costs of implementing prevention strategies in LMICs may be prohibitive, or the skilled labour force to effectively and efficiently conduct highly technical procedures, such as heart valve repair, may take time to develop. Some of these affordability issues could be addresses through a budget-impact analysis conducted alongside CEA/CUA studies.

This review highlights a range of issues in economic analysis for Strep A treatment and prevention modalities. Although this is a scoping study—and it is possible that a small number of relevant papers were not identified—the number of economic evaluations is small relative to the large number of publications evaluating Strep A interventions. This suggests that only a fraction of clinical and implementation studies are considering economic issues, which is depriving decision makers of important decision-making aides. Studies which do have economic evaluation tend to focus on individual clinical-level decision rather than broad public health strategies. This skew in the published literature may lead decision-makers to reach erroneous conclusions about the evidence for different Strep A control modalities and contribute to a narrow focus on clinical rather than public health interventions.

Studies included in this review illustrate the urgent need for a range of better-quality primary data in economic analyses of Strep A prevention and treatment modalities. To guide data collection under constrained resources, a multidisciplinary panel, including experts in infectious diseases, economics, and policy making, could be established to develop a minimum list of costs and consequences to be measured and included in the economic evaluation of Strep A treatment and prevention strategies for several key perspectives. In particular, better data on effectiveness (and efficacy) of a wider range of strategies, improved baseline epidemiology, validation of QALYs in different contexts and cost data are required. While this data is being developed, model-based evaluations should devote greater attention to the results of uncertainty analyses, including identification and discussion of model parameters that are most important for further investigation. In addition, clinical trials of Strep A treatment or prevention strategies would be strengthened by collecting and analysing data on the economic and social benefits of the trialled intervention.

Economic evaluation has the potential to be an important tool for decision makers, but interpretation requires contextual insights of the field. This scoping study identifies that a relatively small number of Strep A studies include economic evaluation and that these are generally focused on specific clinical questions. These studies are often grounded in aging data and opaque or questionable assumptions. There appears to be systematic neglect of economic evaluation of more ambitious approaches to prevent infection, such as vaccination or large-scale public health initiatives. The relatively narrow focus, and limited context, of existing publications on risk-based strategies means that economic evaluation fails to meet it’s potential for informing planning of Strep A control modalities across the spectrum and population affected. This issue is becoming more urgent as progress towards a Strep A vaccine accelerates and the economic case for investment in vaccine development and deployment continues to is explored. This article outlines a range of critical primary data needs which are necessary to address weaknesses in the existing literature and calls for greater clarity and discussion of uncertainties while new data are developed.

## Methods

We conducted a scoping review of the literature for all studies evaluating Strep A treatment or prevention strategies from an economic perspective. A review protocol was developed but not registered at the start of the study and is described here, along with the main findings by following the “Preferred Reporting Items for Systematic Reviews and Meta-Analyses” (PRISMA) guidelines.

### Eligibility criteria

We included all studies published between Jan 1, 2000 and Oct 12, 2022, that measured and valued, in monetary terms, the resources used to treat or prevent Strep A infections or clinical manifestations. We did not include studies on nosocomial infections and on diseases caused by a pathogen other than Strep A (e.g., bacteraemia or toxic shock syndrome due exclusively to *Staphylococcus aureus*). There were no restrictions on study language or country; however, the search terms were in English only. Clinical manifestations arising from Strep A infection included pharyngitis, tonsillitis, peritonsillar abscess (quinsy), impetigo, cellulitis, erysipelas, scarlet fever, necrotising fasciitis, bacteraemia, toxic shock syndrome, sepsis (including maternal sepsis), septic arthritis, osteomyelitis, pneumonia, meningitis, acute rheumatic fever (ARF), rheumatic heart disease (RHD), and acute post-streptococcal glomerulonephritis (APSGN).

Relevant economic study designs included cost-of-illness, cost-effectiveness, cost-utility, cost-benefit, budget-impact, and return-on-investment studies. Studies that did not calculate the intervention cost and compare it to the intervention benefits (clinical or economic) were excluded from the analysis. We did not attempt to calculate cost-effectiveness ratios because any number of measures of effectiveness could be chosen (e.g., cost per disease averted, cost per surgery averted, cost per anaphylaxis reaction averted, cost per days of illness reduced, etc) and interpretation of the ratio is subjective.

### Search strategy

A literature search was conducted using Clarivate Analytics’ Web of Science (WoS) search platform, which includes multiple databases such as the WoS core collection and Medline, and Elsevier’s Embase search platform. Search strings for each database are provided in Supplemental Material 1.

General economic terms and disease terms were restricted to a title search to increase the sensitivity of the search strategy. However, to maximize that chance of identifying all relevant data, references lists of each eligible study were evaluated and hand-searched for studies not identified by the search string.

### Study selection, data extraction process, and data items

Titles and abstracts were screened to identify potential studies. Potential papers meeting the inclusion criteria were sourced in full-text for final review and inclusion or exclusion.

Extracted data included the study’s:

- First author and year of publication
- Country
- Intervention target (e.g., treatment or prevention of a Strep A infection or sequelae) and strategies
- Target population group and age range
- Study design (trial- or model-based evaluation, and type of evaluation (CEA, CBA, CUA))
- Evidence of effectiveness
- Benefits
- Costs
- Evaluation outcomes

### Data synthesis and analysis

**Fig. 2:**
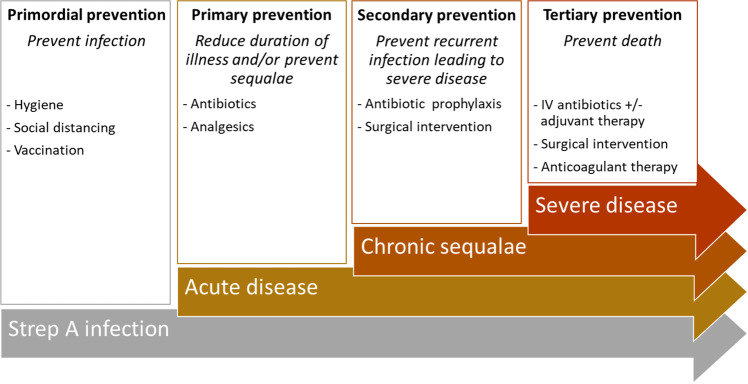
Strep A prevention and treatment stratergies. A framework for the classification of treamtment and prevention stratergies avaiable between Strep A infection and severe outcomes.

We compared and contrasted studies evaluating strategies aimed at similar points in the aetiological pathway between Strep A exposure and death. To do that, we broadened the traditional framework used to categorise prevention strategies for ARF and RHD to cover strategies for all diseases and sequalae caused by Strep A (Fig. 2). In our framework, categories closely but not strictly follow level of care and studies that evaluated strategies from multiple categories were summarised in the most up-stream category (e.g., studies evaluating both primary and secondary prevention strategies were summarised in the primary prevention category). Our categories were defined as follows

1. Primordial prevention: strategies aimed at preventing infection (e.g., hygiene measures, social distancing, and vaccination). Preventing infection prevents acute disease and down-stream outcomes and the need for related down-stream prevention and treatment strategies.
2. Primary prevention: strategies aiming to reduce duration or severity of illness from superficial Strep A infections and/or prevent sequalae, generally by appropriate treatment. Duration of illness can be reduced through antibiotic treatment and relieved through analgesics. Prevention of ARF and other sequelae, such as quinsy and plausibly APSGN, can be achieved by prompt antibiotic treatment of Strep A infection^67,68^. Preliminary review of the literature indicated three subcategories among economic evaluations of primary prevention strategies. They were evaluations of strategies that
  - Reduce duration or severity of illness only
  - Reduce duration of illness and prevent sequalae (e.g., ARF, APSGN, quinsy)
  - Prevent development of autoimmune sequalae only
    Like primordial prevention, primary prevention may prevent down-stream complications and the need for associated prevention and treatment strategies.
3. Secondary prevention: strategies to prevent infection and illness or worsening of an established condition. These strategies typically involve antibiotic prophylaxis and were summarised as those that targeted
  - ARF and direct sequelae
  - Other Strep A diseases
4. Secondary prevention for other Strep A diseases included antibiotic prophylaxis in patients at risk of recurrent cellulitis and among close contacts of people with invasive infection^69–71^. It also included tonsillectomy among people suffering frequently recurrent episodes of tonsillitis. Tertiary prevention: strategies aimed at preventing mortality among those with severe disease, such as cardiovascular complications from RHD or sepsis from invasive infection.

In addition, we assessed the robustness of the evaluations using Drummond et al.’s checklist for assessing economic evaluations^62^.

### Reporting summary

Further information on research design is available in the Nature Research Reporting Summary linked to this article.

## Supplementary information

Supplementary INFO

REPORTING SUMMARY

## Data Availability

The datasets supporting the conclusions of this article are included within the article and its additional files.
