# Supplementary material for: Modalities of group A streptococcal prevention and treatment and their economic justification
Source: NPJ Vaccines. 2023 Apr 22;8:59. doi: 10.1038/s41541-023-00649-3 (PMC10122086; doi:10.1038/s41541-023-00649-3)
Supplement: Supplementary file 1 — Supplementary INFO [file 41541_2023_649_MOESM1_ESM.pdf]

Modalities of group A streptococcal prevention and treatment and their economic justification

Supplementary Methods and Tables

Jeffrey Cannon[1,2]*, Rosemary Wyber[1,3,4]

Affiliations

1. Wesfarmers Centre of Vaccines and Infectious Diseases, Telethon Kids Institute, Nedlands, Western Australia, Australia
2. Department of Global Health and Population, Harvard T.H. Chan School of Public Health, Boston, Massachusetts, USA
3. National Centre for Aboriginal and Torres Strait Islander Wellbeing Research, National Centre for Epidemiology and Population Health, ANU College of Health & Medicine, The Australian National University, Canberra, Australia
4. Adjunct Senior Research Fellow, University of Western Australia, Nedlands, Western Australia, Australia

*Corresponding author
Jeffrey.Cannon@telethonkids.org.au
Telethon Kids Institute
Perth Children's Hospital
15 Hospital Avenue
Nedlands, Western Australia, 6009

Table of Contents

Supplementary Methods 2

Supplementary Table 1. Studies stratified by prevention category 3

Supplementary Table 2. Primordial prevention strategies for disease related to Strep A 6

Supplementary Table 3. Primary prevention strategies for disease related to Strep A - treat acute disease 7

Supplementary Table 4. Primary prevention strategies for disease related to Strep A - treat infection and prevent ARF or other sequelae 13

Supplementary Table 5. Primary prevention strategies for disease related to Strep A – prevent ARF or other sequelae. 16

Supplementary Table 6. Primary prevention strategies for disease related to Strep A with evaluation of secondary and/or tertiary prevention as independent or combined strategies 18

Supplementary Table 7. Secondary and tertiary prevention strategies for disease related to Strep A..... 23

Supplementary Table 8. Secondary prevention strategies for disease related to Strep A – prevent recurrences of diseases other than ARF 25

Supplementary Table 9. Checklist for assessing economic evaluations. 27

Supplementary Methods

Medline and Web of Science

(TI=("group a strep*" OR "Strep* pyogenes" OR pharyngitis OR "sore throat" OR *tonsillitis OR impetigo OR pyoderma OR "skin infection" OR "skin sore" OR "soft tissue" OR cellulitis or erysipelas OR “scarlet fever” OR (rheumatic AND (heart or fever)) OR glomerulonephritis) OR (TI=(sepsis OR septic*emia OR septic OR bacter*emia OR "toxic shock" or fasciitis OR osteomyelitis OR pneumonia OR meningitis) AND TS=(pyogenes or "group A strept*"))) AND

(TS=(cost-benefit OR benefit-cost OR cost-effective* OR cost-minimization OR cost-minimisation OR cost-utility or "cost of illness" OR "return on investment" OR “budget impact” or budget) OR TI=(cost* or economic))

EMBASE

("group a strep*" or "strep* pyogenes" or pharyngitis or "sore throat" or tonsillitis or impetigo or pyoderma or "skin infection" or "skin sore" or "soft tissue" or cellulitis or erysipelas or "scarlet fever" or (rheumatic and (heart or fever)) or glomerulonephritis).ti. or ((sepsis or septic*emia or septic or bacter*emia or "toxic shock" or fasciitis or osteomyelitis or pneumonia or meningitis).ti. and (pyogenes or "group a strep*").mp.) AND

(("cost benefit" or "benefit cost" or "cost effective*" or "cost minimi*ation" or "cost utility" or "cost of illness" or "return on investment" or "budget-impact" or budget).mp. or cost*.ti. or economic.ti.)

Supplementary Table 1. Studies stratified by prevention category

**Study ID**	**Country/region**	**Primordial prevention**	**Primary Prevention**		**Secondary prevention**		**Tertiary prevention**		**Secondary prevention**	
		Prevent infection	Reduce duration of illness	Prevent ARF/other	Prevent recurrent ARF	Prevent clinical/severe RHD	Prevent death		Prevent recurrent tonsillitis	Prevent recurrent cellulitis
Cannon, 2018[17]	Australia	Vaccination								
Burns, 2018[18]	UK		Pharyngitis Rx							
Bura, 2017[19]	Poland		Pharyngitis diagnostics							
Kose, 2016[20]	Turkey		Pharyngitis diagnostics							
Nakhoul, 2013[23]	USA		Pharyngitis diagnostics							
Humair, 2006[21]	Switzerland		Pharyngitis diagnostics							
Portier, 2001[22]	France		Pharyngitis diagnostics							
Anusha, 2019[25]	India		Impetigo Rx							
Alsonso, 2008[24]	Cuba		Impetigo Rx							
Ibrahim, 2019[30]	Australia		Cellulitis; setting							
Li, 2018[31]	USA		Cellulitis; management							
Kameshwar, 2016[32]	Australia		Cellulitis; setting							
Yarbrough, 2015[33]	USA		Cellulitis; management							
Brugha, 2012[34]	UK		Cellulitis; setting							
Vinken, 2003[27]	USA		Cellulitis Rx							
Vinken, 2001[26]	UK		Cellulitis Rx							
Lodise, 2019[28]	USA		SSTI Rx							
Teeres, 2008[29]	Spain		SSTI Rx							
Fraser, 2020[41]	UK		Pharyngitis diagnostics	Pharyngitis diagnostics						

Study ID	Country/region	Primordial prevention	Primary Prevention		Secondary prevention		Tertiary prevention		Secondary prevention	
		Prevent infection	Reduce duration of illness	Prevent ARF/other	Prevent recurrent ARF	Prevent clinical/severe RHD	Prevent death		Prevent recurrent tonsillitis	Prevent recurrent cellulitis
Behnamfar, 2019[38]	Iran		Pharyngitis diagnostics	Pharyngitis diagnostics						
Little, 2014[39]	UK		Pharyngitis diagnostics	Pharyngitis diagnostics (excl. ARF)						
Klepser, 2012[37]	USA		Pharyngitis diagnostics	Pharyngitis diagnostics						
Van Howe, 2006[36]	USA		Pharyngitis diagnostics	Pharyngitis diagnostics						
Neuner, 2003[35]	USA		Pharyngitis diagnostics	Pharyngitis diagnostics						
Irlam, 2013[43]	South Africa			Pharyngitis diagnostics						
Maizia, 2012[46]	France			Pharyngitis diagnostics						
Giraldez-Garcia, 2011[45]	Spain			Pharyngitis diagnostics						
King, 2002[42]	USA			Pharyngitis diagnostics after genetic testing						
Ehrlich, 2002[44]	USA			Pharyngitis diagnostics						
Watkins, 2015[48]	Cuba			Pharyngitis Rx rates	SP adherence					
Nordet, 2008[47]	Cuba			Pharyngitis Rx rates	SP adherence					
Watkins, 2016[49]	African nations			Pharyngitis Rx rates	SP coverage		Access to surgery			
Soudarssanane, 2007[52]	India			Treat	SP		Surgery			

Study ID	Country/region	**Primordial prevention**	**Primary Prevention**		**Secondary prevention**		**Tertiary prevention**		**Secondary prevention**	
		Prevent infection	Reduce duration of illness	Prevent ARF/other	Prevent recurrent ARF	Prevent clinical/severe RHD	Prevent death		Prevent recurrent tonsillitis	Prevent recurrent cellulitis
Coates, 2021[51]	African Union			Pharyngitis Rx rates ; CDR	SP coverage & adherence	SP (echo after 1st ARF), family planning	Surgery, Heart failure management			
Manji, 2013[50]	Developing country (e.g., in sub-Saharan Africa)			Test and treat or prophylaxis for all		Routine echo with SP				
Oetzel, 2019[53]	New Zealand				SP adherence					
Ubels, 2020[55]	Brazil					SP coverage (echo screening)				
Roberts, 2017[54]	Australia					SP coverage (echo screening)				
Zachariah, 2015[56]	Australia					SP coverage (echo screening)				
Uy, 2021[57]	India						Surgery			
Osternmann, 2021[60]	Germany								SilAtro-5-90 adjuvant treatment	
Wilson, 2012[59]	UK								Surgery	
Bhattacharyya, 2002[58]	USA								Surgery	
Mason, 2014[61]	UK									Prophylaxis

ARF, acute rheumatic fever; RHD, rheumatic heart disease; Rx, pharmacological treatment; SP, secondary prophylaxis

Supplementary Table 2. Primordial prevention strategies for disease related to Strep A

Study	Intervention aim and strategies	Country; target population	Study design	Evidence of effectiveness	Benefits	Costs	Outcomes
Cannon, 2018[17]	**Prevention of incident Strep A infection (or clinical disease) and associated sequelae**<br><br>A. Vaccination<br>B. No vaccination	Australia; Infants, 5yo children, and 65yo adults (55yo non-Indigenous Australian adults)	Model-based cost-utility analysis; health sector perspective	Assumed	• Reduce incidence of Strep A diseases<br>• Reduce deaths<br>• Reduce disability-adjusted life-years, comprising acute infection, long-term morbidity, and mortality	• Inpatient admissions<br>• Emergency department episodes<br>• General and specialist consultations<br>• Pharmacy<br>• Outpatient diagnostics<br>• Patient transfers	Cost of childhood vaccination below AU$260-289 for non-Indigenous Australians and AU$897-920 for Indigenous Australians would have a favourable ICER from a health sector perspective.

ICER, incremental cost-effectiveness ratio

Supplementary Table 3. Primary prevention strategies for disease related to Strep A - treat acute disease

Disease/Study	Intervention aim and strategies	Country; target population	Study design	Evidence of effective-ness	Benefits	Costs	Outcomes
**Pharyngitis**							
Burns, 2018[18]	**Reduce duration of sore throat**<br><br>A. A single dose of 10mg oral dexamethasone<br>B. Placebo	UK; adults 18-70 years with acute sore throat and painful swallowing	Trial-based CUA; healthcare services payer perspective	Trial	Improve QALYs based on the EuroQol-five dimensions-five levels (EQ-5D-5L) index.	• Intervention<br>• Visits and telephone calls to the GP<br>• Visits and telephone calls to nurses<br>• Out-of-hours calls and visits<br>• Pharmacy visits<br>• Calls to helpline<br>• Accident and emergency visits<br>• Medication, including prescribed antimicrobials (delayed) and over-the-counter medications	Insufficient evidence to conclude that dexamethasone was cost-effective
Bura, 2017[19]	**Appropriate treatment of pharyngitis**<br><br>A. RADT<br>B. Culture<br>C. Clinical score 3-4<br>D. RADT on clinical score = 2-3 and treat on clinical score = 4	Poland; adult patients aged 18–44 years	Trial-based CEA; perspective not stated	Trial	Improve appropriate antibiotic treatment rates	• Symptomatic treatment<br>• RADT<br>• Culture<br>• Antibiotic course	RADT for all patients and treat positive cases had the lowest cost per appropriate treatment
Kose, 2016[20]	**Appropriate treatment of pharyngitis**	Turkey; patients 3–14 years old who had been	Trial-based CEA; perspective not stated	Trial	Improve appropriate Antibiotic treatment rates	• Antibiotics<br>• RADT	Antibiotic cost and total treatment cost

Disease/Study	Intervention aim and strategies	Country; target population	Study design	Evidence of effective-ness	Benefits	Costs	Outcomes
	**(including choice of antibiotic)**<br><br>A. RADT<br>B. Empirical treatment	diagnosed with pharyngitis and who had not received antibiotics for 7 days before inclusion.					per patient was lower for routine RADT compared to routine empirical treatment
Nakhoul, 2013[23]	**Appropriate treatment of pharyngitis**<br><br>A. DNA backup after false RADT<br>B. No Backup after false RADT	USA; all patients aged 18 years and older visiting a generalist physician with a diagnosis of acute pharyngitis	Post-hoc CEA of an observational trial; perspective not stated	Trial	Correction of false-negative RADT	DNA probe	The additional expense of routine backup testing by DNA probe in adults is not justified
Humair, 2006[21]	**Appropriate treatment of pharyngitis**<br><br>A. Symptomatic treatment<br>B. RADT all<br>C. RADT on clinical score = 2-3) and empirical treatment on clinical score = 4<br>D. Empirical Treatment on clinical score = 3-4<br>E. Culture all	Switzerland; adult patients older than 15 years with pharyngitis and least 2 of 4 Centor criteria	Trial-based CEA; perspective not stated	Trial	Improve appropriate antibiotic treatment rates	• RADT<br>• Culture<br>• Antibiotics	Routine RADT had the lowest cost-effectiveness ratio
Portier, 2001[22]	**Appropriate treatment of pharyngitis**	France; treatment of	Trial-based CBA;	Trial	Reduce net cost	• Symptomatic treatment<br>• Antibiotics	The mean total treatment cost

Disease/Study	Intervention aim and strategies	Country; target population	Study design	Evidence of effective-ness	Benefits	Costs	Outcomes
	A. RADT all<br>B. No RADT	pharyngitis in adults (> 25 years of age). Patients presenting with rhinopharyngitis or pharyngitis not suggesting a streptococcal etiology were excluded	perspective not stated			• Complementary exams<br>• Consult<br>• RADT<br>• Leave days	per episode of pharyngitis was lower for routine RADT compared to usual practice
**Impetigo**							
Anusha, 2019[25]	**Impetigo treatment**<br><br>A. Fusidic acid<br>B. Mupirocin	India; impetigo patients aged 1-30 years	Trial-based CEA; perspective not stated	Trial	Improve cure rate	Treatment drug	Fusidic acid is cost-effective compared to mupirocin
Alfonso, 2008[24]	**Impetigo treatment**<br><br>A. Ozonized oil<br>B. Mupirocin cream	Cuba; impetigo patients aged <15 years	Trial-based CEA; perspective not stated	Trial	Improve cure rate	Treatment drug	Ozonized oil is more effective and less costly than mupirocin cream
**Cellulitis**							
Ibrahim, 2019[30]	**Reduce treatment failure rate and improve QALYs.**<br><br>A. Intravenous ceftriaxone at home<br>B. Intravenous flucloxacillin in hospital	Australia; Children aged 6 months to 18 years who had presented to an emergency department (ED) with moderate or severe uncomplicated cellulitis	Trial-based CUA and CEA; Healthcare and family perspectives	Trial	• Reduce treatment failure<br>• Improve QALYs (from the Child Health Utility 9D (CHU9D) questionnaire)	Healthcare-related:<br>• Salaries<br>• Admission process<br>• ED<br>• Pathology<br>• Pharmacy<br>• Other<br>• Transport<br><br>Family:	Intravenous ceftriaxone at home was more effective and less costly from both perspectives

Disease/Study	Intervention aim and strategies	Country; target population	Study design	Evidence of effective-ness	Benefits	Costs	Outcomes
						• Absence from paid and from unpaid work<br>• Expenses during treatment	
Li, 2018[31]	**Reduce cellulitis misdiagnosis**<br><br>A. Early dermatology consultation<br>B. Usual care	USA; patients presenting to an emergency department with an initial diagnosis of cellulitis	Model-based CBA; perspective not stated	Trial and literature	Reduce:<br>• Antibiotic consumption<br>• Inpatient length of stay<br>• Complications	• Dermatology services<br>• Inpatient days	Net cost savings for early dermatology consultation
Kameshwar, 2016[32]	**Reduce duration of treatment for lower-limb cellulitis**<br><br>A. Transfer to hospital in the home (HITH)<br>B. Usual (inpatient) care	Australia; patients admitted for lower-limb cellulitis for 24 or more hors	Trial-based CBA; institutional perspective	Trial	• Reduce duration of treatment	• Direct patient costs (medical, nursing and allied health wages, ward consumables, theatre costs, drugs, anaesthetics, pathology and imaging)<br>• Overheads (utilities, cleaning, medical records, finance, administration, security, information technology and engineering)	Duration of treatment was longer for HTIH compared to impatient management but costs were statistically similar
Yarbrough, 2015[33]	**Reduce unnecessary treatment for cellulitis**	USA; cellulitis patients 18 years or older admitted to the	Trial-based CEA; perspective not stated	Trial	• Reduce antibiotic consumption	• Pharmacy<br>• Laboratory<br>• Imaging<br>• Facility	The evidence-based care pathway was

Disease/Study	Intervention aim and strategies	Country; target population	Study design	Evidence of effective-ness	Benefits	Costs	Outcomes
	A. Evidence-based care pathway<br>B. Usual care	emergency department or hospital			• Reduce diagnostic tests<br>• Reduce 30-day readmission rate		less costly for similar effect
Brugha, 2012[34]	**Prevent complications of preseptal cellulitis**<br><br>A. IV antibiotic treatment by ambulatory care<br>B. IV antibiotic treatment by inpatient care	UK; children <16 years presenting to Emergency Department with signs and symptoms of preseptal cellulitis	Trial-based CBA; perspective not stated	Trial	• Reduced duration of treatment	• Inpatient admission<br>• High complexity ED attendance	Ambulatory treatment is less costly for a similar duration of treatment
Vinken, 2003[27]	**Successful empiric treatment of cellulitis (given methicillin resistant S aureus)**<br><br>A. IV oxacillin (with an option to switch to oral dicloxacillin)<br>B. IV vancomycin<br>C. IV linezolid (with an option to switch to oral).	USA; patients hospitalised for cellulitis	Model-based CMA; hospital perspective	Literature	• Treatment success	• Antibiotic course<br>• Ward stay<br>• Intensive care	Linezolid is likely to be less costly compared with vancomycin at all resistance rates and with oxacillin when the risk of infection with methicillin-resistant pathogens is greater than 18.7%
Vinken, 2001[26]	**Successful empiric treatment of cellulitis (given**	UK; patients hospitalised for cellulitis	Model-based CMA; hospital perspective	Literature	• Treatment success	• Antibiotic course<br>• Day treatment<br>• Day stay	Initiating treatment with linezolid was more effective

Disease/Study	Intervention aim and strategies	Country; target population	Study design	Evidence of effective-ness	Benefits	Costs	Outcomes
	**methicillin resistant S aureus)**<br><br>A. Initiate treatment with IV linezolid<br>B. Standard care					• General ward stay<br>• Intensive care unit stay	and less costly than with flucloxacillin
**Skin and soft tissue infections (SSTIs)**							
Lodise, 2019[28]	**SSTI treatment success in outpatient settings**<br><br>A. Single dose oritavancin<br>B. Multidose vancomycin	USA; patients who were >18 years old and treated in an outpatient setting with oritavancin or vancomycin	Retrospective trial-based CEA; perspective not stated	Trial	• lower 30-day hospital admission rates	• Inpatient admission<br>• ED visits<br>• Outpatient medical services<br>• Outpatient pharmacy prescription costs	Oritavancin had a statistically similar cost to vancomycin but was more effective
Terres, 2008[29]	**Cost-minimisation of SSTIs treatment**<br><br>A. Tigecycline (100 mg initially, followed by 50 mg IV every 12 hours)<br>B. Vancomycin plus aztreonam (1000/2000 mg IV every 12 hours)	Spain; adult patients hospitalised with complicated SSTI	Retrospective trial-based CMA; national health system perspective	Trial	• NA	• Inpatient admission and readmission<br>• Ward days<br>• Intensive care unit hours<br>• Antibiotic treatment<br>• Pathology and diagnostic tests	Treating complicated skin and soft tissue infections with tigecycline is cheaper than with ancomycin plus aztreonam

GP, general practice; RADT, rapid antigen detection test; ICER, incremental cost-effectiveness ratio; CEA, cost-effectiveness analysis; CUA, cost-utility analysis; CBA, cost-benefit analysis; CMA, cost-minimisation analysis; QALYs, quality-adjusted life-years; IV, intravenous; CDR, clinical decision rule

Supplementary Table 4. Primary prevention strategies for disease related to Strep A - treat infection and prevent ARF or other sequelae

Study	Intervention aim and strategies	Country; target population	Study design	Evidence of effectiveness	Benefits	Costs	Outcomes
Fraser, 2020[41]	**Reduce duration of illness and risk of complications**<br><br>A. CDR + RADT (several manufacturers)<br>B. CDR (usual care)	UK; people aged ≥ 5 years presenting with sore throat and a FeverPAIN score of 4 or 5 points or a Centor score of 3 or 4 points	Model-based CUA (stratified by age, children or adults); NHS/Personal Social Services perspective	Systematic review and meta-analyses	• QALDs, comprising treated and untreated infection, penicillin-induced rash, Quincy, penicillin-induced anaphylaxis (sepsis), and ARF.	• GP consultation<br>• Antibiotics<br>• Pain relief (paracetamol)<br>• Throat culture<br>• Admission for sepsis, abscess, or ARF<br>• RADT (cost of test plus GP time)	Usual care
Behnamfar, 2019[38]	**Reduce duration of illness and risk of complications**<br><br>A. Treat all<br>B. Treat none<br>C. RADT<br>D. Culture<br>E. RADT + culture all<br>F. RADT + culture -ve RADT	Iran; people presenting to a primary healthcare clinic with pharyngitis	Model-based CUA and CBA; perspective unclear.	Not reported (values nor source)	• QALDs, comprising PTA, anaphylaxis, adverse reaction, ARF, untreated GAS, and death	• PTA, anaphylaxis, adverse reaction, 'acute renal failure', APSGN	RADT all
Little, 2014[39]	**Reduce duration of illness and risk of quinsy and other complications (excl. ARF)**<br><br>A. CDR (FeverPAIN); score <2 = no rx, score 2-3=delayed	UK; aged ≥ 3 years and acute sore throat	Trial-based CEA and CUA with modelled long-term outcomes; National Health	Trial	• 'Symptom severity score'<br>• QALYs, based on EQ5D scores measured during the trial	• Visit to GP<br>• GP home visit<br>• Per minute of GP time<br>• Visit to NP<br>• Antibiotic course and prescribing cost<br>• Visit to A&E	CDR is less costly and more effective

Study	Intervention aim and strategies	Country; target population	Study design	Evidence of effectiveness	Benefits	Costs	Outcomes
	rx; score 4+ = immediate rx.<br>B. Usual care (delayed rx; fill script if symptoms worsen or persist after 3-5 days)<br>C. RADT if CDR 3+ (delayed rx if CDR=2)		Services perspective			• Overnight hospital stay	
Klepser, 2012[37]	**Reduce duration of illness and risk of complications**<br><br>A. Phyicisan observation<br>B. physician treatment of all<br>C. physician throat culture<br>D. physician RADT, and throat culture on negative RADT<br>E. physician RADT only<br>F. nurse practitioner in a walk-in clinic performing an RADT on all patients<br>G. Pharmacist RADT on all patients (and dispensing antibiotics)	USA; adults seeking treatment for pharyngitis	Model-based CUA; perspective not stated	Literature	• QALDs, comprising duration of illness, penicillin-induced rash, anaphylaxis, quinsy, uncomplicated ARF, RHD, and death	• RADT<br>• Calling in prescription<br>• Culture<br>• Penicillin<br>• Penicillin-induced rash<br>• Anaphylaxis<br>• Uncomplicated ARF<br>• GP visit<br>• Walk-in clinic visit<br>• Pharmacist visit	Strep A treatment provided by a pharmacist was the most cost-effective treatment

Study	Intervention aim and strategies	Country; target population	Study design	Evidence of effectiveness	Benefits	Costs	Outcomes
Van Howe, 2006[36]	**Reduce duration of illness and risk of complications**<br><br>A. Treat none<br>B. Treat all<br>C. RADT<br>D. Culture<br>E. RADT then culture<br>F. Clinical decision rule	USA; children presenting with pharyngitis	Model-based CUA; societal and payer perspectives	Literature	• QALDs, comprising quinsy, ARF, death, penicillin reaction, duration of illness	• Quinsy<br>• Penicillin<br>• Cephalosporin<br>• ARF<br>• Culture<br>• Death<br>• Penicillin reaction<br>• RHD<br>• Follow-up telephone call<br>• Parental wages	Societal: throat culture based on Medicaid rates; RADT based on private insurance rates<br><br>Payer: treat none
Neuner, 2003[35]	**Reduce duration of illness and risk of complications**<br><br>A: Observation without testing or treatment<br>B: Empirical treatment with penicillin<br>C: Throat culture<br>D: RADT followed by culture to confirm negative test<br>E: RADT alone.	USA; adults presenting with pharyngitis	Model-based CUA; *societal* perspective	Literature	• QALDs, comprising duration of illness, penicillin-induced rash and anaphylaxis, quinsy, uncomplicated ARF, RHD, and death.	• Tests (point-of-care and throat culture)<br>• Culture result notification<br>• Calling in a prescription<br>• Penicillin<br>• Penicillin-induced rash<br>• Anaphylaxis<br>• PTA<br>• ARF	Culture least costly and most effective

GP, general practice; RADT, rapid antigen detection test; ICER, incremental cost-effectiveness ratio; CEA, cost-effectiveness analysis; CUA, cost-utility analysis; CBA, cost-benefit analysis; QALYs, quality-adjusted life-years; QALDs, quality-adjusted life-days

Supplementary Table 5. Primary prevention strategies for disease related to Strep A – prevent ARF or other sequelae.

Study	Intervention aim and strategies	Country; target population	Study design	Evidence of effectiveness	Benefits	Costs	Outcomes
Irlam, 2013[43]	Prevent ARF and RHD<br><br>A. Treat all<br>B. Culture<br>C. CDR<br>D. CDR & culture -ve<br>E. Treat none	South Africa; children between the ages of 3 to 15 years who present to an urban primary care clinic with a complaint of sore throat and no history of ARF	Model-based CUA; societal perspective	Literature	QALYs, comprising ARF, RHD without surgery, RHD post-surgery, ARF, anaphylaxis, quinsy, and rash	• Outpatient visit at primary care clinic + out of pocket<br>• penicillin-induced rash and anaphylaxis<br>• Throat culture<br>• Outpatient visit at hospital<br>• Quinsy<br>• ARF<br>• Secondary prophylaxis<br>• RHD	Treat those with a CDR score of 2+ is cost-effective (US$ 136 per QALY)
Maizia, 2012[46]	Prevent suppurative complications (not explicitly defined)<br><br>A. Observation only [no treatment]<br>B. CDR<br>C. RADT<br>D. Culture<br>E. CDR >=2, then RADT<br>F. RADT + culture -ve<br>G. Treat all	France; children and adults presenting to primary clinics	Model-based CEA; health insurance perspective	Literature	Risk of suppurative complications	• Medical consultation<br>• RADT<br>• Culture<br>• Antibiotic<br>• Serious allergic reactions	The use of the RADT alone had the best cost-effectiveness ratio in both adults and children: 970 Euros in children and at 903 Euros in adults per suppurative complication avoided.
Giraldez-Garcia, 2011[45]	Prevent complications<br><br>A. Treat all<br>B. CDR<br>C. RADT	Spain; patients between the ages of 2 and 14 years who consult with a primary care physician due to	Model-based CEA; Spanish National	Literature	• Suppurative complications<br>• ARF<br>• Unnecessary treatments	• GP visit<br>• Phone call<br>• RADT<br>• Culture<br>• Penicillin	CDR + RADT was cost-effective in preventing complications (from Strep A or penicillin)

Study	Intervention aim and strategies	Country; target population	Study design	Evidence of effectiveness	Benefits	Costs	Outcomes
	D. Culture<br>E. RADT + culture<br>F. CDR + RADT	pharyngitis symptoms	Health Service perspective		• Allergic reactions<br>• Deaths	• Azithromycin<br>• Reaction<br>• Suppurative complications<br>• ARF	compared to culture
King, 2002[42]	Prevent ARF<br><br>A. Screen all children at birth for genetic susceptibility to RF and provide monthly or daily antibiotic prophylaxis in susceptible, otherwise usual care<br>B. Usual care for all	USA; 2000 birth cohort	Model-based CUA; societal perspective	Assumed	QALYs	• Genetic testing<br>• Prophylaxis<br>• Pharyngitis management<br>• ARF admission and long-term management<br>• Heart failure-related care<br>• Endocarditis<br>• Surgery	Genetic testing was cost-effective compared to usual care
Ehrlich, 2002[44]	Prevent ARF<br><br>A. Treat none<br>B. Treat all<br>C. RADT<br>D. Culture<br>E. RADT + culture	USA; paediatric population aged 5 to 17 presenting with sore throat	Model-based CEA; societal perspective	Literature	• RHD<br>• ARF<br>• Suppurative complications	• Culture<br>• Antibiotic treatment and reaction<br>• Suppurative complications<br>• ARF	RADT was cost-effective

GP, general practice; RADT, rapid antigen detection test; ICER, incremental cost-effectiveness ratio; CEA, cost-effectiveness analysis; CUA, cost-utility analysis; CBA, cost-benefit analysis; CMA, cost-minimisation analysis; QALYs, quality-adjusted life-years; CDR, clinical decision rule

Supplementary Table 6. Primary prevention strategies for disease related to Strep A with evaluation of secondary and/or tertiary prevention as independent or combined strategies

Study	Intervention aim and strategies	Country; target population	Study design	Evidence of effectiveness	Benefits	Costs	Outcomes
Watkins, 2015[48]	**Prevent incident ARF/RHD and worsening or prevalent RHD**<br><br>A. Intervention - Improve health-seeking behaviours for sore throat patient adherence to penicillin prophylaxis<br>B. Do nothing	Cuba; children aged 5 – 24 years	Post-hoc CUA of a population-wide program	Literature (Nordet, 2008)	DALYs	• Hospitalisation<br>• Long-term prophylaxis and specialist care<br>• Prevention program	The intervention was net cost-saving and more effective than usual care
Nordet, 2008[47]	**Prevent incident ARF/RHD and worsening or prevalent RHD**<br><br>A. Do nothing<br>B. Improve health-seeking behaviours for sore throat patient adherence to penicillin prophylaxis	Cuba; school children	Post-hoc CEA of a population-wide program	Analysis of population-level data	• Incidence of ARF and RHD<br>• Prevalence of ARF and RHD	• Total cost of ARF and RHD care<br>• Intervention	The cost of care for ARF/RHD significantly reduced for a relatively minor cost for the intervention
Watkins, 2016[49]	**Prevent incident ARF and RHD**<br><br>Scaling up:<br>A. Primary prevention	African nations; population aged 5-25 years	Model-based CUA; health system perspective	Literature and assumptions	DALYs	• ARF hospitalisation<br>• Remission<br>• RHD<br>• Cardiovascular complications<br>• Interventions	Primary prevention is cost saving (comparator not stated)<br>Secondary preventions is cost effective

Study	Intervention aim and strategies	Country; target population	Study design	Evidence of effectiveness	Benefits	Costs	Outcomes
	(pharyngitis treatment rate)<br>B. Secondary prevention (prophylaxis compliance rates)<br>C. Heart valve surgery						(comparator not stated)
Soudarssanane, 2007[52]	**Prevent incident ARF and RHD**<br><br>A. Primary prevention<br>B. Secondary prevention<br>C. Tertiary prevention	India; people aged 5-15 years	Model-based CBA; government and community perspectives	Assumed	• Productivity from improved life expectancy<br>• Reduced costs of upstream prevention strategies	Primary prevention<br>• GP consultation<br>• Throat culture<br>• Patient travel<br>• Pain relievers<br>• Antibiotics<br>• Indirect costs<br>Secondary prevention<br>• Hospital admission<br>• Lost wages<br>• Diagnostic services<br>• Antibiotics<br>• Pain relievers<br>• Prophylaxis (9 years)<br>• Patient travel<br>• Specialist follow up<br>• Community cost<br>• Social cost (school failures)<br>• Incidental costs<br>Tertiary prevention	Primary prevention and secondary prevention resulted in positive net benefits, and primary prevention had the highest net benefit amount

Study	Intervention aim and strategies	Country; target population	Study design	Evidence of effectiveness	Benefits	Costs	Outcomes
						• Surgery<br>• Post-surgery care<br>• Lost wages<br>• Patient travel<br>• Specialist follow up<br>• Cost of not implementing secondary prevention<br>• Rheumatic fever admissions and management<br>• Incidental costs	
Coates, 2021[51]	**Prevent incident ARF/RHD and worsening or prevalent RHD**	African Union; population between 2021 and 2030	Model-based CBA; health system perspective	Literature	Monetary value of reduced morbidity and mortality	Primary prevention<br>• Public health campaign<br>• Outpatient consultation<br>• Antibiotics<br>• Provider education and evaluation<br>• Community health worker training, equipment, and administrative costs<br>Secondary prevention<br>• Outpatient visit<br>• Antibiotics	Scale up of secondary prevention and secondary and tertiary care interventions resulted in a positive net benefit, and was negative for primary prevention

Study	Intervention aim and strategies	Country; target population	Study design	Evidence of effectiveness	Benefits	Costs	Outcomes
						• Patient transport<br>• Echocardiography evaluation<br>• Provider education and evaluation<br>• Public health campaign<br>• Handheld ultrasound equipment<br>• Training<br>• Other supplies<br>Tertiary prevention<br>• Heart failure management (tradeable and non-tradeable costs)<br>• Handheld ultrasound equipment<br>• INR machine<br>• Training<br>• Other supplies<br>• Valve surgery<br>• Patient transport<br>• Post-surgical management (tradeable and non-tradeable costs)<br>Other<br>• ARF admission	

Study	Intervention aim and strategies	Country; target population	Study design	Evidence of effectiveness	Benefits	Costs	Outcomes
Manji, 2013[50]	**Prevent incident ARF and/or RHD**<br><br>A. Culture (50% seek treatment) and treatment<br>B. Antibiotic prophylaxis for all regardless of clinical history<br>C. Echo and SP<br>D. Do nothing	Developing country (e.g., in sub-Saharan Africa); people between 5 and 21 years-old for intervention, 5-70 years for modelled outcomes	Model-based CUA; perspective not stated	Literature and assumptions	QALYs, comprising Strep throat, antibiotic side effects, RHD	• Throat swab<br>• Lab analysis of swab<br>• Wage for swab<br>• Antibiotics<br>• Ab side effects<br>• Ab prophylaxis<br>• Echocardiography<br>• Wage for cardiography<br>• Echo machine<br>Direct and indirect costs of RHD	ECHO is cost-effective compared to doing nothing; strategies A and B are not cost-effective compared to echo.

GP, general practice; RADT, rapid antigen detection test; ICER, incremental cost-effectiveness ratio; CEA, cost-effectiveness analysis; CUA, cost-utility analysis; CBA, cost-benefit analysis; CMA, cost-minimisation analysis; QALYs, quality-adjusted life-years; IV, intravenous; CDR, clinical decision rule

Supplementary Table 7. Secondary and tertiary prevention strategies for disease related to Strep A

Category/Study	Intervention aim and strategies	Country; target population	Study design	Evidence of effectiveness	Benefits	Costs	Outcomes
Secondary prevention							
Oetzel, 2019[53]	Improve patient compliance to secondary prophylaxis<br><br>A. Incentives for adherence<br>B. Usual care	New Zealand; patients on prophylaxis management	Trial-based CEA; public provider perspective	Trial	Improved adherence (recommended schedule of BPG injections)	• Incentives<br>• BPG injections<br>• Follow up phone calls	ICER subject to decision maker's acceptability threshold
Ubels, 2020[55]	Prevent worsening of RHD<br><br>A. Routine screening<br>B. Usual care	Brazil; 11-year-old children	Model-based CUA; public perspective	Assumed	Reduce DALYs	• Screening<br>• RHD treatment<br>• Surgery	Screening is cost-effective compared to usual care
Roberts, 2017[54]	Prevent worsening of RHD<br><br>A. Routine screening<br>B. Usual care	Australia; remote Indigenous children aged 5 to 12 years	Model-based CUA; health sector perspective	Assumed	Reduce:<br>• RHD severity at diagnosis<br>• Heart failures<br>• Surgeries<br>• Deaths<br>• DALYs, comprising acute infection, long-term sequalae, and death	• ARF and RHD diagnosis and long term management<br>• Surgery<br>• Screening	Cost-effective is cost-effective compared to usual care if RHD can be detected ≥2 years earlier by screening
Zachariah, 2015[56]	Prevent worsening of RHD<br><br>A. Routine screening<br>B. Usual care	Australia; 11-year-old children	Model-based CUA; societal perspective	Assumed	Improved QALYs	• Screening<br>• Primary care for subclinical RHD<br>• RHD diagnosis and long term management	Screening is likely less costly and more effective compared to usual care

Category/Study	Intervention aim and strategies	Country; target population	Study design	Evidence of effectiveness	Benefits	Costs	Outcomes
						• Surgery<br>• Management post-valve surgery<br>• Lost wages	
Tertiary							
Uy, 2021[57]	**Prevent worsening of heart disease or death**<br><br>A. Valve repair<br>B. Mechanical valve replacement (MVR-M)<br>C. Bioprosthetic valve replacement (MVR-B)<br>D. Usual care (mix of A-C)	India; patients aged 20 years and older with rheumatic mitral valve disease	Model-based CUA; public payer perspective	Literature	• Reduced reoperations<br>• Reduced cardiovascular complications<br>• Improved QALYs	• Surgery<br>• Post-surgery management<br>• Cardiovascular complications	Repair was less costly and more effective than usual care and MVR-M. The ICER for MVR-B compared to repair was cost-effective at a threshold of India's per-capita gross domestic product

ICER, incremental cost-effectiveness ratio; CEA, cost-effectiveness analysis; CUA, cost-utility analysis; CBA, cost-benefit analysis; QALYs, quality-adjusted life-years; DALYs, disability-adjusted life-years

Supplementary Table 8. Secondary prevention strategies for disease related to Strep A – prevent recurrences of diseases other than ARF

Category/Study	Intervention aim and strategies	Country; target population	Study design	Evidence of effectiveness	Benefits	Costs	Outcomes/ recommendations
Osternmann, 2021[60]	**Prevent recurrent throat infections**<br><br>A. SilAtro-5-90 adjuvant therapy<br>B. Usual care	Germany; patients with suspected moderate recurrent tonsillitis	Model-based CEA; societal perspective	Literature	Averted throat infections	• SilAtro-5-90<br>• Tonsillectomy<br>• Antibiotics<br>• GP<br>• Productivity loss<br>• Career time<br>• Travel time	Adolescents/Adults >12-years-old: ICER subject to decision maker's acceptability threshold<br><br>Children <= 6-12-years-old: adjuvant therapy is less costly and more effective than usual care
Wilson, 2012[59]	**Prevent recurrent pharyngitis**<br><br>A. Tonsillectomy<br>B. Medical therapy	UK; children aged 4 to 15 years with recurrent sore throats	Trial-based CEA; perspective not stated	Trial	• Reduced recurrent sore throat and associated treatment<br>• Improved quality of life (PedsQL Pediatric Quality of Life inventory score)	*Not reported*	"..., tonsillectomy can save up to 8 sore throats at a reasonable cost..."
Bhattacharyy, 2002[58]	Tonsillectomy	USA; adults with chromic tonsillitis	(retrospective) Trial-based CEA; perspective not stated	Trial	• Improved quality of life (Glasgow Benefit Inventory)<br>Reduced:<br>• Antibiotic consumption	• Physician consultations<br>• Antibiotics<br>• Productivity loss<br>• Tonsillectomy	Tonsillectomy "...results in significant improvement in quality of life, decreases health-care utilization, and diminishes the economic burden of chronic tonsillitis"

Category/Study	Intervention aim and strategies	Country; target population	Study design	Evidence of effectiveness	Benefits	Costs	Outcomes/ recommendations
					• Productivity loss<br>• Physician consultations		
Mason, 2014[61]	Prevent recurrent cellulitis (of the leg)	UK	Multi trial-based CUA; public health and societal perspectives	Trials	• Reduction in recurrent infections<br>• Improved QALYs (EuroQoL EQ-5D-3L)	• GP consultation<br>• Nurse visits<br>• Inpatient stays<br>• Outpatient visits<br>• Days of work<br>• Antibiotics	"Antibiotic prophylaxis reduces cellulitis recurrence by nearly a third but is not associated with a significant increase in costs."

GP, General Practice; CEA, cost-effectiveness analysis; CUA, cost-utility analysis; QALYs, quality-adjusted life-years; ICER, incremental cost-effectiveness ratio

Supplementary Table 9. Checklist for assessing economic evaluations.[62]

	Checklist Items										
Study	Well defined question	Description of competing strategies	Effectiveness established	Costs and consequences identified for each strategy	Costs and consequences measured accurately	Credible costs and consequences valuation	Appropriate discounting	Incremental analysis	Uncertainty analyses	Presentation/discussion of study results include all issues of concern	Total
Cannon, 2018[17]	1	1	0	1	1	1	1	1	1	0	8
Burns, 2018[18]	1	1	1	1	1	1	1	1	1	1	10
Bura, 2017[19]	0	1	1	0	1	0	1	0	0	0	4
Kose, 2016[20]	0	1	1	0	1	0	1	0	0	0	4
Nakhoul, 2013[23]	0	1	1	0	1	1	1	1	0	1	7
Humair, 2006[21]	0	1	1	0	1	0	1	0	1	1	6
Portier, 2001[22]	0	1	1	0	1	1	1	0	0	0	5
Anusha, 2019[25]	0	1	1	0	1	0	1	0	0	0	4
Alsonso, 2008[24]	0	1	1	0	0	1	1	1	1	0	6
Ibrahim, 2019[30]	1	1	1	1	1	1	1	1	1	1	10
Li, 2018[31]	0	1	1	0	1	1	1	0	0	1	6
Kameshwar, 2016[32]	1	1	1	1	1	1	1	0	1	1	9
Yarbrough, 2015[33]	0	0	1	0	0	1	1	0	1	0	4
Brugha, 2012[34]	0	1	1	0	0	1	1	0	1	0	5
Vinken, 2003[27]	1	1	1	1	1	1	1	0	1	1	9
Vinken, 2001[26]	1	1	1	1	1	1	1	0	1	1	9
Lodise, 2019[28]	0	1	1	0	1	0	1	0	1	1	6
Teeres, 2008[29]	1	1	1	1	1	1	1	1	1	1	10
Fraser, 2020[41]	1	1	1	1	1	1	1	1	1	1	10
Behnamfar, 2019[38]	1	1	0	1	1	0	1	1	1	1	8
Little, 2014[39]	1	1	1	1	1	1	1	1	1	1	10
Klepser, 2012[37]	0	1	1	0	1	1	1	1	1	1	8
Van Howe, 2006[36]	1	1	1	1	1	1	1	1	1	0	9

Neuner, 2003[35]	1	1	1	1	1	1	1	1	1	1	10
Irlam, 2013[43]	1	1	1	1	1	1	1	1	1	1	10
Maizia, 2012[46]	1	1	1	1	1	1	1	1	1	1	10
Giraldez-Garcia, 2011[45]	1	1	1	1	1	1	1	1	1	1	10
King, 2002[42]	1	1	0	1	1	1	1	1	1	0	8
Ehrlich, 2002[44]	1	1	0	1	1	1	1	1	1	1	9
Watkins, 2015[48]	1	1	0	1	1	1	1	1	1	1	9
Nordet, 2008[47]	0	0	0	0	0	0	0	0	0	0	0
Watkins, 2016[49]	1	1	1	1	0	0	1	1	1	1	8
Soudarssanane, 2007[52]	0	1	0	0	1	0	0	1	0	0	3
Coates, 2021[51]	1	1	0	1	1	1	1	0	1	1	8
Manji, 2013[50]	0	0	0	0	1	0	1	1	1	0	4
Oetzel, 2019[53]	0	0	1	0	1	1	1	1	0	1	6
Ubels, 2020[55]	1	1	0	1	1	1	1	1	1	1	9
Roberts, 2017[54]	1	1	0	1	1	1	1	1	1	1	9
Zachariah, 2015[56]	1	1	0	1	1	0	1	1	1	0	7
Uy, 2021[57]	1	1	1	1	1	1	1	1	1	1	10
Osternmann, 2021[60]	1	1	1	1	1	1	1	1	1	1	10
Wilson, 2012[59]	0	1	1	0	1	1	1	1	1	1	8
Bhattacharyya, 2002[58]	0	1	1	0	1	1	1	0	1	1	7
Mason, 2014[61]	1	1	1	1	1	1	1	1	1	1	10
Number (%) of evaluations with full criteria	26 (59%)	40 (91%)	32 (73%)	26 (59%)	39 (89%)	33 (75%)	42 (95%)	29 (66%)	35 (80%)	30 (68%)	

References (copied verbatim from the main text for numeric consistency)

1 Hoy, W. E., White, A. V., Dowling, A., Sharma, S. K., Bloomfield, H., *et al.* Post-streptococcal glomerulonephritis is a strong risk factor for chronic kidney disease in later life. *Kidney International* **81**, 1026-1032 (2012).

2 Oda, T. & Yoshizawa, N. Factors Affecting the Progression of Infection-Related Glomerulonephritis to Chronic Kidney Disease. *Int J Mol Sci* **22**, 905 (2021).

3 Beaton, A., Kamalembo, F. B., Dale, J., Kado, J. H., Karthikeyan, G., *et al.* The American Heart Association's Call to Action for Reducing the Global Burden of Rheumatic Heart Disease: A Policy Statement From the American Heart Association. *Circulation* **142**, e358-e368 (2020).

4 Fleming-Dutra, K. E., Hersh, A. L., Shapiro, D. J., Bartoces, M., Enns, E. A., *et al.* Prevalence of Inappropriate Antibiotic Prescriptions Among US Ambulatory Care Visits, 2010-2011. *Jama-Journal of the American Medical Association* **315**, 1864-1873 (2016).

5 Grigoryan, L., Haaijer-Ruskamp, F. M., Burgerhof, J. G. M., Mechtler, R., Deschepper, R., *et al.* Self-medication with antimicrobial drugs in Europe. *Emerging Infectious Diseases* **12**, 452-459 (2006).

6 Wyber, R., Noonan, K., Halkon, C., Enkel, S., Cannon, J., *et al.* Ending rheumatic heart disease in Australia: the evidence for a new approach. *Medical Journal of Australia* **213 Suppl 10**, S3-s31 (2020).

7 Marijon, E., Mirabel, M., Celermajer, D. S. & Jouven, X. Rheumatic heart disease. *The Lancet* **379**, 953-964 (2012).

8 Barron, J., Turner, R., Jaeger, M., Adamson, W. & Singer, J. Comparing the use of intravenous antibiotics under the medical benefit with the use of oral antibiotics under the pharmacy benefit in treating skin and soft tissue infections. *Managed care (Langhorne, Pa.)* **21**, 44-52 (2012).

9 Boyler, P. A., Humair, J., Revaz, S. A. & Stalder, H. A cost-effectiveness analysis of recommended strategies for acute pharyngitis. *Journal of General Internal Medicine* **17**, 135-136 (2002).

10 Dall, L., Peterson, S., Simmons, T. & Dall, T. Outpatient treatment of CAP cellulitis generates cost savings. *Drug Benefit Trends* **16**, 21-22+24-28+32 (2004).

11 Eron, L. J., King, P. & Marineau, M. Antibiotic selection and hospital discharge of patients with cellulitis. *Infections in Medicine* **21**, 381-+ (2004).

12 Perone, N. & Humair, J. P. Diagnosis and management of pharyngitis. *Revue medicale suisse* **3**, 286-290 (2007).

13 Yang, C. C. & Pan, C. Y. Evaluation of cost-effectiveness for prophylactic antibiotic in recurrent cellulitis of lower limb. *Journal of Internal Medicine of Taiwan* **22**, 352-362 (2011).

14 Baumann, I. Outcome after tonsillectomy in chronic tonsillitis. [German]. *Hno* **53**, 405-407 (2005).

15 Love, B. L. & Kehr, H. Management of complicated skin and soft tissue infections in hospitalized patients. *U.S* **Pharmacist. 32(4)**, HS5-HS12 (2007).

16 Tang Girdwood, S. C., Sellas, M. N., Courter, J. D., Liberio, B., Tchou, M. J., *et al.* Improving the transition of intravenous to enteral antibiotics in pediatric patients with pneumonia or skin and soft tissue infections. *Journal of Hospital Medicine* **15**, 9-15 (2020).

17 Cannon, J. W., Jack, S., Wu, Y., Zhang, J., Baker, M. G., *et al.* An economic case for a vaccine to prevent group A streptococcus skin infections. *Vaccine* **36**, 6968-6978 (2018).

18 Burns, R. M., Wolstenholme, J., Jawad, S., Williams, N., Thompson, M., *et al.* Economic analysis of oral dexamethasone for symptom relief of sore throat: the UK TOAST study. *Bmj Open* **8**, e019184 (2018).

19 Bura, M., Michalak, M., Chojnicki, M., Padzik, M. & Mozer-Lisewska, I. Moderate and severe pharyngitis in young adult inhabitants of Poznan, western Poland. *Family Medicine & Primary Care Review* **19**, 12-17 (2017).
20 Kose, E., Kose, S. S., Akca, D., Yildiz, K., Elmas, C.*, et al.* The Effect of Rapid Antigen Detection Test on Antibiotic Prescription Decision of Clinicians and Reducing Antibiotic Costs in Children with Acute Pharyngitis. *Journal of Tropical Pediatrics* **62**, 308-315 (2016).
21 Humair, J. P., Revaz, S. A., Bovier, P. & Stalder, H. Management of acute pharyngitis in adults - Reliability of rapid streptococcal tests and clinical findings. *Archives of Internal Medicine* **166**, 640-644 (2006).
22 Portier, H., Peyramond, D., Boucot, I., Grappin, M., Boibieux, A.*, et al.* Assessing applicability of guidelines on management of pharyngitis in adults in general practice. *Medecine et Maladies Infectieuses* **31**, 396-402 (2001).
23 Nakhoul, G. N. & Hickner, J. Management of Adults with Acute Streptococcal Pharyngitis: Minimal Value for Backup Strep Testing and Overuse of Antibiotics. *Journal of General Internal Medicine* **28**, 830-834 (2013).
24 Alfonso, K., Collazo, M., Fernandez, M. & Ballagas, C. Cost-efficacy Analysis of Topical Ozonized Oil versus Mupirocin Cream in the Treatment of Impetigo. *Latin American Journal of Pharmacy* **27**, 512-518 (2008).
25 Anusha Rani, M. V., Bhuvaneshwari, E. & Venkatakrishna, A. Comparison of efficacy and cost-effectiveness of topical fusidic acid and topical mupirocin in the treatment of impetigo. *National Journal of Physiology, Pharmacy and Pharmacology* **9**, 1225-1229 (2019).
26 Vinken, A., Li, Z., Balan, D., Rittenhouse, B., Wilike, R.*, et al.* Economic evaluation of linezolid, flucloxacillin and vancomycin in the empirical treatment of cellulitis in UK hospitals: a decision analytical model. *The Journal of hospital infection* **49 Suppl A**, S13-24 (2001).
27 Vinken, A. G., Li, J. Z., Balan, D. A., Rittenhouse, B. E., Willke, R. J.*, et al.* Comparison of linezolid with oxacillin or vancomycin in the empiric treatment of cellulitis in US hospitals. *American journal of therapeutics* **10**, 264-274 (2003).
28 Lodise, T. P., Palazzolo, C., Reksc, K., Packnett, E. & Redell, M. Comparisons of 30-Day Admission and 30-Day Total Healthcare Costs Between Patients Who Were Treated With Oritavancin or Vancomycin for a Skin Infection in the Outpatient Setting. *Open Forum Infectious Diseases* **6** (2019).
29 Terres, C. R. Pharmacoeconomic analysis of the treatment of complicated skin and soft tissue infections with tigecycline or the combination of vancomycin and aztreonam in Spain. [Spanish]. *Pharmacoeconomics - Spanish Research Articles* **5**, 23-33 (2008).
30 Ibrahim, L. F., Huang, L., Hopper, S. M., Dalziel, K., Babl, F. E.*, et al.* Intravenous ceftriaxone at home versus intravenous flucloxacillin in hospital for children with cellulitis: a cost-effectiveness analysis. *Lancet Infectious Diseases* **19**, 1101-1108 (2019).
31 Li, D. G., Xia, F. D., Khosravi, H., Dewan, A. K., Pallin, D. J.*, et al.* Outcomes of Early Dermatology Consultation for Inpatients Diagnosed With Cellulitis. *Jama Dermatology* **154**, 537-543 (2018).
32 Kameshwar, K., Karahalios, A., Janus, E. & Karunajeewa, H. False economies in home-based parenteral antibiotic treatment: a health-economic case study of management of lower-limb cellulitis in Australia. *Journal of Antimicrobial Chemotherapy* **71**, 830-835 (2016).
33 Yarbrough, P. M., Kukhareva, P. V., Spivak, E. S., Hopkins, C. & Kawamoto, K. Evidence-Based Care Pathway for Cellulitis Improves Process, Clinical, and Cost Outcomes. *Journal of Hospital Medicine* **10**, 780-786 (2015).
34 Brugha, R. E. & Abrahamson, E. Ambulatory Intravenous Antibiotic Therapy for Children With Preseptal Cellulitis. *Pediatric Emergency Care* **28**, 226-228 (2012).

35 Neuner, J. M., Hamel, M. B., Phillips, R. S., Bona, K. & Aronson, M. D. Diagnosis and Management of Adults with Pharyngitis: A Cost-Effectiveness Analysis. *Annals of Internal Medicine* **139**, 113-122 (2003).

36 Van Howe, R. S. & Kusnier, L. P. Diagnosis and management of pharyngitis in a pediatric population based on cost-effectiveness and projected health outcomes. *Pediatrics* **117**, 609-619 (2006).

37 Klepser, D. G., Bisanz, S. E. & Klepser, M. E. Cost-Effectiveness of Pharmacist-Provided Treatment of Adult Pharyngitis. *American Journal of Managed Care* **18**, E145-E150 (2012).

38 Behnamfar, Z., Shahkarami, V., Sohrabi, S., Aghdam, A. S. & Afzali, H. Cost and effectiveness analysis of the diagnostic and therapeutic approaches of group A Streptococcus pharyngitis management in Iran. *Journal of Family Medicine and Primary Care* **8**, 2942-2949 (2019).

39 Little, P., Hobbs, F. R., Moore, M., Mant, D., Williamson, I.*, et al.* PRImary care Streptococcal Management (PRISM) study: in vitro study, diagnostic cohorts and a pragmatic adaptive randomised controlled trial with nested qualitative study and cost-effectiveness study. *Health Technol Assess* **18** (2014).

40 Lloyd, A. & Pickard, A. S. The EQ-5D and the EuroQol Group. *Value Health* **22**, 21-22 (2019).

41 Fraser, H., Gallacher, D., Achana, F., Court, R., Taylor-Phillips, S.*, et al.* Rapid antigen detection and molecular tests for group A streptococcal infections for acute sore throat: systematic reviews and economic evaluation. *Health Technology Assessment* **24**, 1-+ (2020).

42 King, C. H., Fischler, D. F. & Gerkin, R. D. Will genetic testing alter the management of disease caused by infectious agents? A cost-effectiveness analysis of gene-testing strategies for prevention of rheumatic fever. *Clinical Infectious Diseases* **34**, 1491-1499 (2002).

43 Irlam, J., Mayosi, B. M., Engel, M. & Gaziano, T. A. Primary Prevention of Acute Rheumatic Fever and Rheumatic Heart Disease With Penicillin in South African Children With Pharyngitis A Cost-Effectiveness Analysis. *Circulation-Cardiovascular Quality and Outcomes* **6**, 343-351 (2013).

44 Ehrlich, J. E., Demopoulos, B. P., Daniel, K. R., Ricarte, M. C. & Glied, S. Cost-effectiveness of treatment options for prevention of rheumatic heart disease from group A streptococcal pharyngitis in a pediatric population. *Preventive Medicine* **35**, 250-257 (2002).

45 Giraldez-Garcia, C., Rubio, B., Gallegos-Braun, J. F., Imaz, I., Gonzalez-Enriquez, J.*, et al.* Diagnosis and management of acute pharyngitis in a paediatric population: a cost-effectiveness analysis. *European Journal of Pediatrics* **170**, 1059-1067 (2011).

46 Maizia, A., Letrilliart, L. & Colin, C. Diagnostic strategies for acute tonsillitis in France: a cost-effectiveness study. *Presse medicale (Paris, France : 1983)* **41**, e195-203 (2012).

47 Nordet, P., Lopez, R., Duenas, A. & Sarmiento, L. Prevention and control of rheumatic fever and rheumatic heart disease: the Cuban experience (1986-1996-2002). *Cardiovascular journal of Africa* **19**, 135-140 (2008).

48 Watkins, D. A., Mvundura, M., Nordet, P. & Mayosi, B. M. A Cost-Effectiveness Analysis of a Program to Control Rheumatic Fever and Rheumatic Heart Disease in Pinar del Rio, Cuba. *Plos One* **10**, e0121363 (2015).

49 Watkins, D., Lubinga, S. J., Mayosi, B. & Babigumira, J. B. A Cost-Effectiveness Tool to Guide the Prioritization of Interventions for Rheumatic Fever and Rheumatic Heart Disease Control in African Nations. *Plos Neglected Tropical Diseases* **10**, e0004860 (2016).

50 Manji, R. A., Witt, J., Tappia, P. S., Jung, Y., Menkis, A. H.*, et al.* Cost-effectiveness analysis of rheumatic heart disease prevention strategies. *Expert Review of Pharmacoeconomics & Outcomes Research* **13**, 715-724 (2013).

51 Coates, M. M., Sliwa, K., Watkins, D. A., Zuhlke, L., Perel, P.*, et al.* An investment case for the prevention and management of rheumatic heart disease in the African Union 2021-30: a modelling study. *Lancet Global Health* **9**, E957-E966 (2021).

52 Soudarssanane, M. B., Karthigeyan, M., Mahalakshmy, T., Sahai, A., Srinivasan, S.*, et al.* Rheumatic fever and rheumatic heart disease : Primary prevention is the cost effective option. *Indian Journal of Pediatrics* **74**, 567-570 (2007).
53 Oetzel, J. G., Lao, C., Morley, M., Penman, K., Child, M.*, et al.* Efficacy of an incentive intervention on secondary prophylaxis for young people with rheumatic fever: a multiple baseline study. *Bmc Public Health* **19**, 385 (2019).
54 Roberts, K., Cannon, J., Atkinson, D., Brown, A., Maguire, G.*, et al.* Echocardiographic Screening for Rheumatic Heart Disease in Indigenous Australian Children: A Cost-Utility Analysis. *Journal of the American Heart Association* **6**, e004515 (2017).
55 Ubels, J., Sable, C., Beaton, A. Z., Nunes, M. C. P., Oliveira, K. K. B.*, et al.* Cost-Effectiveness of Rheumatic Heart Disease Echocardiographic Screening in Brazil: Data from the PROVAR+ Study. *Global Heart* **15**, 18 (2020).
56 Zachariah, J. P. & Samnaliev, M. Echo-based screening of rheumatic heart disease in children: a cost-effectiveness Markov model. *Journal of Medical Economics* **18**, 410-419 (2015).
57 Uy, J., Ketkar, A. G., Portnoy, A. & Kim, J. J. Cost-utility analysis of heart surgeries for young adults with severe rheumatic mitral valve disease in India. *International Journal of Cardiology* **338**, 50-57 (2021).
58 Bhattacharyya, N. & Kepnes, L. J. Economic benefit of tonsillectomy in adults with chronic tonsillitis. *Annals of Otology Rhinology and Laryngology* **111**, 983-988 (2002).
59 Wilson, J. A., Steen, I. N., Lock, C. A., Eccles, M. P., Carrie, S.*, et al.* Tonsillectomy: A Cost-Effective Option for Childhood Sore Throat? Further Analysis of a Randomized Controlled Trial. *Otolaryngology-Head and Neck Surgery* **146**, 122-128 (2012).
60 Ostermann, T., Park, A. L., De Jaegere, S., Fetz, K., Klement, P.*, et al.* Cost-effectiveness analysis for SilAtro-5-90 adjuvant treatment in the management of recurrent tonsillitis, compared with usual care only. *Cost Effectiveness and Resource Allocation* **19**, 60 (2021).
61 Mason, J. M., Thomas, K. S., Crook, A. M., Foster, K. A., Chalmers, J. R.*, et al.* Prophylactic Antibiotics to Prevent Cellulitis of the Leg: Economic Analysis of the PATCH I & II Trials. *Plos One* **9**, e82694 (2014).
62 Drummond, M. F., Sculpher, M. J., Stoddart, G. L., Torrance, G. W., Drummond, M.*, et al.* *Methods for the economic evaluation of health care programmes*. Fourth edition. edn, (Oxford University Press, 2015).
63 Wyber, R., Wade, V., Anderson, A., Schreiber, Y., Saginur, R.*, et al.* Rheumatic heart disease in Indigenous young peoples. *Lancet Child Adolesc Health* **5**, 437-446 (2021).
64 Parks, T., Smeesters, P. R. & Steer, A. C. Streptococcal skin infection and rheumatic heart disease. *Current Opinion in Infectious Diseases* **25**, 145-153 (2012).
65 Thomas, S., Bennett, J., Jack, S., Oliver, J., Purdie, G.*, et al.* Descriptive analysis of group A Streptococcus in skin swabs and acute rheumatic fever, Auckland, New Zealand, 2010–2016. *The Lancet Regional Health - Western Pacific* **8**, 100101 (2021).
66 Wyber, R., Noonan, K., Halkon, C., Enkel, S., Cannon, J.*, et al.* Ending rheumatic heart disease in Australia: the evidence for a new approach. *Medical Journal of Australia* **213**, S3-S31 (2020).
67 Lorgelly, P. K., Lawson, K. D., Fenwick, E. A. & Briggs, A. H. Outcome measurement in economic evaluations of public health interventions: a role for the capability approach? *Int J Environ Res Public Health* **7**, 2274-2289 (2010).
68 Spinks, A., Glasziou, P. P. & Del Mar, C. B. Antibiotics for treatment of sore throat in children and adults. *Cochrane Database of Systematic Reviews* (2021).
69 Bateman, E., Mansour, S., Okafor, E., Arrington, K., Hong, B. Y.*, et al.* Examining the Efficacy of Antimicrobial Therapy in Preventing the Development of Postinfectious Glomerulonephritis: A Systematic Review and Meta-Analysis. *Infect Dis Rep* **14**, 176-183 (2022).

70 de Almeida Torres, R. S., dos Santos, T. Z., Torres, R. A., Petrini, L. M., Burger, M.*, et al.* Management of Contacts of Patients With Severe Invasive Group A Streptococcal Infection. *J Pediatric Infect Dis Soc* **5**, 47-52 (2016).
71 Moore, D. L., Allen, U. D. & Mailman, T. Invasive group A streptococcal disease: Management and chemoprophylaxis. *Paediatr Child Health* **24**, 128-129 (2019).
72 Raff, A. B. & Kroshinsky, D. Cellulitis: A Review. *JAMA* **316**, 325-337 (2016).
